# Anniversary Meeting at Leicester Royal Infirmary

**Published:** 1914-03-21

**Authors:** Henry Burdett


### HOSPITAL TREATMENT AND NATIONAL INSURANCE.

### Anniversary Meeting at Leicester Royal Infirmary.

ADDRESS BY SIR HENRY BURDETT.

The one hundred and forty-first anniversary meeting of Leicester Royal Infirmary was held in the new Children's Hospital on Wednesday, last week. Sir Henry Burdett presided and delivered an address on "Hospital Treatment and National Insurance." There was a large attendance of governors and subscribers, and among the numerous distinguished persons on the platform were Sir Edward Wood, chairman of the hospital board, the Mayor, Councillor J. Russell Fears, Mr. T. Cope, chairman of the County Council, Sir Arthur Hazlerigg, Mr. S. F. Stone, Dr. Pratt, and Mr. C. J. Bond.

In moving the adoption of the report and balance sheet for the year, Sir Edward Wood, on behalf of the board, thanked Sir Henry for his attendance, and said they knew him as a great expert who would tell them frankly what he thought. They had had a record year of in-patients, only seven short of 4,000, and their expenditure grew with their work. Their income was £25,102, which was the largest they had ever had, but there was a deficit of £72. They had now a Children's Hospital, and next year they would require £29,000. He wanted particularly to refer to the Hospital Saturday Fund. The contributions from the working men and women had reached last year, the first year of the Insurance Act, £15,638, which was a splendid record. They had only 2,000 annual subscriptions in a town of 500,000 inhabitants, and he asked that the general class of subscriptions should be increased. The hospital deserved their help, and they wanted an increase of £4,400 next year.

Mr. C. J. Bond, the vice-chairman, seconded, and urged the claim of the pathological department, which needed enlargement. The immediate problem was how to bring modern and accurate methods of diagnosis and treatment within the reach primarily of the honorary staffs of the voluntary hospitals, and secondly—which was the great problem of the Insurance Act—within the reach of panel practitioners. In the hospital at Cambridge, which did not deal with half the number of patients they had in Leicester, a movement was on foot for equipping the clinical laboratory and for providing a pathological department for investigating the blood and body secretions, so as to place the best modern methods at the disposal of the Cambridge medical staff. That was what was needed at Leicester.

### Sir Henry Burdett's Address.

Sir Edward Wood, Ladies, and Gentlemen,—It is my privilege and pleasure to support the adoption of the report. I have known the Leicester Infirmary since 1868, when I paid it my first visit, in the days when Dr. Marriott was a young man very anxious about his future, and most anxious that you should have an ophthalmic department of the first rank in connection with this institution. So that my purview and knowledge of this institution extend longer perhaps than the lifetime of some here present, and have represented altogether a period of nearly fifty years. I was very glad, indeed, when I received an invitation to preside at this meeting, because I felt particularly the responsibility which rests upon those who have to finance voluntary hospitals in view of the very uncertain and most unsatisfactory outlook which the medical services of this country have to face to-day. We have got a national system of insurance. That national system is one which I welcome; I had a proposal on a non-contributory basis which many years ago was thought to be sound and good, and yet I myself am glad that we have a compulsory system. It is all very well for politicians to talk about changing this and changing that, but the bodies and the lives and the welfare of great masses of the people of this country are now interwoven with national insurance.

Before I go into this question I wish naturally to address myself, first of all, to the Royal Infirmary and its present position, both in relation to other hospitals and its general efficiency, and also in regard to these great changes which the country has to face. Finally, I wish to examine the question of adequate medical treatment. There can be no doubt—and I have come here fresh from four years' work of inspection of the hospitals of England—that Leicester Infirmary occupies a very high position, and that it holds a unique position. Leicester is unique—and it is a happy thing for the people of this town and county that it is so—in the fact that although it has not a medical school in the ordinary meaning of the term, yet it is one of the most valued centres of graduate education that these islands contain. You have here in this Infirmary a remarkable state of affairs. There are no students in the ordinary sense of the word, and all the work of this hospital has to be done by resident medical officers and nurses. It used to be thought impossible to carry on an instituti[illegible] of this type adequately on those lines, with all the surgical treatment and new developments in treatment which you would find in all the best organised hospitals. But you have been blessed for many years, in Miss Rogers, with a woman of exceptional character and exceptional knowledge. I am glad to find that you have found so worthy a successor to her as you have in Miss Vincent. I am glad to know that you have recognised what you owe to the nurses: your Nurses' Home is, on the whole, as complete and as attractive as any Home in the country. Well, then, there came the question of the medical care, and to effect that it was essential that you should have a large staff of resident medical officers. I was glad to find that the attractiveness and the importance of this hospital to the younger members of the medical profession are so widely recognised throughout the schools, and that you have here representatives of many of the chief hospitals, and that even the great University of Edinburgh has discovered Leicester. With that Scotch shrewdness which makes them so wonderfully successful in the world I find several Scotchmen of the first rank amongst your resident medical officers. That you may take from me as proof positive of the excellence of this institution as well as of the splendid field of medical and surgical practice which this hospital affords. Now the policy of your hospital during the last twelve years, under Sir Edward Wood, has been the policy which every hospital that is successful must follow.

### The Humbug of Closing Beds.

I see in the papers to-day that there is a complaint from Loughborough that the hospital in that town is languishing because it cannot get money, and the condition of those who are responsible for its administration—I speak quite frankly on these occasions—the condition of mind of those people who are responsible for this want of money and this want of efficiency is described in the papers in this way, that the committee say, and the papers say, that if they don't get more money they have only one course before them. And what course is that? To close the beds. Well, now, ladies and gentlemen, if a hospital committee tell me when they want money that they are going to close beds, I come to the conclusion that that committee know nothing whatever about hospital administration. And the reason is quite simple, because if they were to close half the beds they would still want money; they would not save half the cost of those beds; if their beds cost £60 each, and they closed twenty beds, they would not save £1,200 a year by the transaction at all, because the really heavy charge of a hospital is the central administration; and the closing of beds is a humbug and a fraud, which has often been used to tickle the imagination of people who are very difficult to win as givers. It may have succeeded in the past, but, if so, it succeeded on false premises. Now, what that hospital wants is what you were fortunate enough to find twelve years ago in the person of Sir Edward Wood. I am very glad, incidentally, to have seen that account of the Loughborough Hospital, because I heard yesterday, in going into the affairs of this institution, about the new administrative scheme which your responsible committee have had the wisdom to put before you—namely, a conference of administrative bodies of the town and county, and the linking up by the representation of all those authorities on the board of governors, with this hospital as the central institution in the county of Leicester. Now, that scheme is one which I commend to the committee at Loughborough. All these authorities have been asked to send representatives to the hospital board, and by acting in co-operation and inter-communication to unite to reduce the expenditure by devoting economical consideration in obtaining the supplies of all the hospitals in the county of Leicester. But Loughborough have not come in. Wake up, Loughborough! That is my message to them.

### The Leicester Board's Policy.

Now, what has the board's policy done? When I was here in 1909 you were badly in want of £3,000 a year more income, and when I went into your figures yesterday I found that you had taken this to heart, and that as a fact you have not only got the extra £3,000 a year, but the increase is, in fact, £4,660. That is creditable to the administration of this hospital, and creditable to the people of Leicester. I am perfectly certain that no man or woman who goes to a hospital once can fail to realise that the only unselfish good that the created can render to the Creator is praise, and the form of their praise will be by personal service in the days of health for the blessings which they enjoy. This question of the attitude of the healthy individual towards the sick is one of the most important of all the moral things that we can understand. There is nothing in the world so blessed as acquiring knowledge of the privilege to render service by praise in personal service for the sick in the days of health. I have 400,000 people associated to-day in the League of Mercy who recognise that principle, and practise it habitually every day of their lives. And I hope in the town of Leicester that all you people who contribute to the hospitals won't be content to give your money. Money is a good thing, but when we are prosperous enough to have more than sufficient to pay the requirements of life, what is the good of our money to us? Really what we give is often a mere matter of a book entry, and I don't think it is going to be much to our credit in the days that are to come. Don't let us have any more difficulty about getting the remainder of the money that is required for the Leicester Infirmary, and let me at least have the satisfaction of knowing on December 31, if I am alive, that my coming down here was not altogether useless, but that you hastened up to give that you might show praise to Almighty God for the blessings of health.

You want in this hospital certain things. When I came down here before you wanted £3,000 a year, and you have got £4,660. I thank you. I am pleased. You also wanted a mortuary, and you have not got a mortuary. I want a mortuary because I think a great hospital like this should be an education for the whole people, and one of the things a hospital should teach is reverence and respect for the dead. I am very strong about that. I assure you the influence which comes from a centre like this and spreads throughout the community is absolutely good. I want this hospital to hurry up and come into line upon this point.

Leicester should also hurry up and get that pathological department established quickly. It is momentous to you in regard to diagnosis and treatment, and affects everyone in this place, and the sooner you get it the better, as Mr. Bond has wisely said.

### Two Novel Departures.

I have told you about your new policy of linking up, and I have told you also about the enlargement of the board by representation of the administrative bodies. These are two most important and novel departures. You have taken the devil to your bosom because it has always been held with regard to the maintenance of voluntary hospitals that anything to do with the municipality or any other authority supported out of the rates was absolute destruction and death. I agree with the administrators of this hospital that the time has come when any feeling or idea of that sort has to be wiped out. What we have got to get is a perfect system of hospital administration and medical relief for the whole country, and that can only be got by inter-communication and co-operation. You know, as the report indicates, that the position of affairs at the present moment created by national insurance, so far as the ordinary hospitals are concerned, is this: The hospitals were in a great difficulty, because Mr. Lloyd George had claimed all through that there should be adequate medical benefit, and the Bill provided nothing of the kind. You cannot have adequate medical benefit without hospital treatment. There was nothing, except inferentially, which gave any opportunity of getting any money to assist the hospitals to help the insured, and that was all taken away in the first amending Act which was passed. So we are left to-day in the position of the insured person wanting this care, and depending on the voluntary hospitals for it, and finding the position is that national insurance in this country would be in liquidation to-day if it were not for the voluntary hospitals. Now, as to our unit. You are in the position that at this hospital you are undertaking to treat the patients that are sent to you by the panel doctors. I hope I shall not hurt anybody's feelings when I say that amongst the cases that are sent to the hospital are many that ought not to be sent. I do not think they would be sent if the daily papers published a list of those sent by the panel doctors to the hospitals in every district and what was found to be the matter with them. However, we get these cases, and we then have to do the costly part of our work, which is providing in-patient treatment. You cannot get more than a pint into a pint pot, and the immediate effect is that the voluntary hospitals which are well managed try to keep about 10 per cent. of their beds vacant, because a great emergency may arise when they may want fifty or sixty beds and will be filled to overflowing. That is part of the inconvenience of national insurance, and it has produced a state of things which has become intolerable and dangerous. It is intolerable in the sense that the waiting-list is growing and growing, and that is a thing which no wise administrator can tolerate, because if they need in-patient care, being bread-winners, every day that care is withheld you are imperilling the future of the individual. The consequence is that we shall have to find means to supply the necessary beds to provide for the adequate treatment of all these patients, and the treatment of them at once.

### The Need for Co-operation.

Well, many people say that the remedy is State hospitals. Here, again, we have the impatient utterance of ignorance, absolute ignorance. The State hospital system is one governed and dominated by science. The very basis of our hospital system is that the first consideration, and not the last, is the patient. The voluntary system of hospitals is one of the glories of the world. Another thing—financially you cannot have a State system. A system of State hospitals, as was proved before the Poor-Law Commission, means £20,000,000 more on the expenditure of this country at once, and it may ultimately be increased to £30,000,000. Where are you going to find the money? It is all very well, but you cannot do it. We come back, therefore, to the fact that voluntary hospitals must be supported. We have got first of all to find out what are the full needs of the population. We do not know at present how many beds are required, and we cannot tell how many are required. On the waiting-lists there are a large number, but if, then, there are a large number of beds required, there is no doubt about this, that there are a large number of beds available which we have not yet brought into use. That brings us into the need for inter-communication and co-operation between the authorities and the taking of a careful return of the beds that are available, so that we can bring them into occupation. What we want to do in this country is to come to some arrangement under national insurance which will give to insured persons adequate medical treatment in the shape of hospital care as and when they require it. In doing that we ought to do it on a basis which will maintain the voluntary system of hospitals. This is quite a business arrangement, and very simple. The State comes to the voluntary hospital, as it must come after next July, and says: We want so many beds for our insured persons. The hospitals say: We are voluntary institutions, we cannot make a profit out of this business, but we will oblige you by having these cases, and you will pay us *pro rata* the whole cost entailed upon the hospitals by the admission and treatment of each insured person, plus 10 per cent. for the remuneration of the medical staff. If you do this we would then set aside some wards, which might be called State wards or insured wards, into which those people would come, and the Insurance Commissioners—they ought to do it, and not the local committee—would enter into an arrangement to pay the *pro rata* rate.

### Provision of Pay Pavilions.

That would not be all, because we would still have to provide the hospital care which is necessary for other sections of the community. That can be done by having pavilions erected which will provide for each class that will pay—those who can pay in full. The only thing you have got to do is that it may be administered by this hospital, but there must be a separate entrance, so that doctors can come in from outside without interfering with or having anything to do with the administration of the hospital. These doctors will be the patients' own doctors, and will be paid for in the ordinary way. There is in this matter the question of capital. How are you to get the money for the buildings? That money will have to be raised, I think, by pledging the credit of the country, as has been done before in other matters. You advance to the hospitals, they finding the site, an adequate sum to build these pavilions at interest at the rate of 4 per cent.—1 per cent. for the sinking fund and 3 per cent. interest—so that it would not actually cost the country anything at all, and they would have for their security a first charge on the contributions for the inmates of these buildings. In that way, worked out quite simply and regularly, we should be able to extend our system and carry it through effectually so far as hospital care is concerned, at an increased cost which is relatively so small as to place it well within the means of the funds which would be available under the Government. This being so, I come back to Leicester, and what do I find?

### Beginnings at Leicester.

I find that by anticipation you have recognised the price of the requirements that I have been talking about, and you have set to work already to provide many of the things that are necessary. For instance, you have institutions in Leicester which enable all kinds of people to get hospital care and to pay for it, according to their means, and this is a very remarkable fact. It is one of considerable hope and considerable importance. You can go to the Faire Hospital and pay 25s. a week, and there you can receive as good accommodation in the hospital sense as any man can require. I go on to the maternity hospital, and there they have a system of attending to mothers, and they are working it out there. It is true that they are only cottages, and it is not a modern up-to-date institution, but every woman has a room and this room she is accustomed to, and I think on the whole they are very comfortable. They also pay, and there is no reason why that should not be quite successful. The fact that it exists proves the need for what I am advocating. Then you have another hospital—the Highfield Hospital—which takes patients in and gives them the right to pay what they can. In Leicester the infirmary came first, and the cottage hospital movement has been put under. But you have gone to all the hospitals there are in the county and you have asked them to come into co-operation with you, so that you may send out to them cases which will benefit by the country air while they send to you cases which require more skilled treatment or surgical aid than they are able to give in the smaller institutions. The infirmary is in the centre of the system, and it is quite worth while seeing how that can be carried out.

Now, I have one thing more to say in conclusion. I am immensely struck—and I have got to say it in his presence—with the exceedingly good fortune and the providential guidance over this institution which is characterised by two things. One is the advent of Sir Edward Wood twelve years ago, when, mind you, in many respects this hospital was in a position of extreme peril. It wanted money, it wanted rebuilding, it wanted modernising, and if it did not do all these things it could never maintain its position, and must have gone under. Now, in Sir Edward Wood you have a gentleman who all the time has his mind at work seeing how he can work this institution financially and otherwise. The results of Sir Edward Wood's works are manifest and they will live for ever. The second point is that in the person of Mr. Bond you have a member of the medical profession who I have had the honour and pleasure of having amongst my friends for many years. He is a man who carried out the same principle in the exercise of his profession that Sir Edward Wood has done in the building up of this institution.

The Poor-Law Infirmary.

I have spent some very long days here, and I have told you what I think about it. I have not mentioned the Poor-Law infirmary, an institution which I spent a whole afternoon inspecting. I can only say of it to-day that it is not a hospital; it is a building which has been erected at great cost, which as a building is highly efficient in every particular, except that it is not a hospital, because it is not administered as a hospital. I have had a communication from the chairman of the committee of that Poor-Law infirmary asking me to make a report upon it, and I shall do so in due course. I believe what is wrong is entirely due to want of knowledge, and not from any absence of desire to do what is right. I have much pleasure in supporting the adoption of the report.

A hearty vote of thanks having been accorded him for his address, Sir Henry, in responding, said: I omitted in my remarks to mention the Children's Hospital. You can do anything you like for the children of Leicester, but if you are going to do it in connection with the public authorities I hope you will make a business-like arrangement with them, in order that this institution shall not be put to a heavy charge for doing work which does not rightly appertain to it. I think before I sit down I might very properly congratulate you extremely upon your secretary and house governor, Mr. Harry Johnson. He is one of the men who stand out pre-eminent amongst the men of intelligence and ability who have been associated with the progress of the voluntary hospitals. The way in which he turns out his literature, the way in which he puts forward his annual reports, the spirit and energy he puts into things are very strong evidences to me of his high efficiency and his extreme value. I hope you pay him adequately, and that you will help him. Mr. Johnson is a good man, and it is worth your while to pay him well. I hope you will not only get that money for the pathological block, but that you will by April 20 have raised the £3,000 you require to complete the building fund of the Children's Hospital.

